# Treatment of Hepatic Abscess

**Published:** 1904-03-05

**Authors:** 


### TREATMENT OF HEPATIC ABSCESS.

Mr. James Cantlie[1] discusses the operative treatment of abscess of the liver, and gives the notes of 10 cases upon which he operated by trocar and cannula and siphon drainage. He emphasises the importance of immediate exploration of the liver with an aspirating needle if there be a suspicion of hepatic abscess. Pus may be reached by the first puncture, but if not the liver should be punctured in at least six places before coming to the conclusion that there is no abscess. When blood flows into the syringe it is a good plan to draw off a few ounces to relieve congestion. Mr. Cantlie has never observed dangerous hæmorrhage follow such punctures of the liver, although cases have been recorded where this has happened. To obviate the risk of wounding the inferior vena cava he acts on the anatomical observation that the inferior vena cava at the level of the liver is situated at practically equal distances from the surface along a line leading from the middle of the body in front to the angle of the ribs on the right side—the area in which a needle must be introduced in searching for pus. In a patient whose body measures 32 inches in circumference at this level the inferior vena cava is $4\frac{1}{2}$ inches from the surface, so that a needle may be introduced without danger to a distance of $3\frac{1}{2}$ inches. In stouter people, of course, the needle may be introduced further. The importance of being assured of the comparative safety of exploring the liver with an aspirating needle is obvious when it is considered that a man may have a large liver abscess and yet have no pain, cough, rise of temperature, and so forth to show its existence, and that if left alone such an abscess may burst into the lung or the bowel, with dire consequences. Mr. Cantlie advocates the treatment by trocar and cannula and siphon drainage in all cases of deep-seated hepatic abscess—and in every case the pus is deep-seated in the early stages. The advantages which this method has for medical men practising in the tropics, very probably far removed from any well-equipped hospital, do not need to be pointed out.

1 Jour. of Tropical Medicine, Feb. 1.

